# Disease Burden Due to Herpes Zoster among Population Aged ≥50 Years Old in China: A Community Based Retrospective Survey

**DOI:** 10.1371/journal.pone.0152660

**Published:** 2016-04-07

**Authors:** Yan Li, Zhijie An, Dapeng Yin, Yanmin Liu, Zhuoying Huang, Jianfang Xu, Yujie Ma, Qiufeng Tu, Qi Li, Huaqing Wang

**Affiliations:** 1 National immunization program, Chinese center for disease control and prevention, Beijing, China; 2 Immunization program department, Shanghai municipal center for disease control and prevention, Shanghai, China; 3 Immunization program department, Danyang county center for disease control and prevention, Zhenjiang, Jiangsu, China; 4 Immunization program department, Heilongjiang provincial center for disease control and prevention, Haerbin, Heilongjiang, China; 5 Immunization program department, Jiangxi provincial center for disease control and prevention, Nanchang, Jiangxi, China; 6 Immunization program department, Hebei provincial center for disease control and prevention, Shijiazhuang, Hebei, China; Rega Institute for Medical Research, BELGIUM

## Abstract

**Objective:**

To understand the disease burden due to Herpes Zoster (HZ) among people aged ≥50 years old in China and provide baseline data for future similar studies, and provide evidence for development of herpes zoster vaccination strategy.

**Methods:**

Retrospective cohort study was conducted in 4 townships and one community. A questionnaire was used to collect information on incidence and cost of HZ among people aged ≥ 50 years old.

**Results:**

The cumulative incidence rate was 22.6/1,000 among people aged ≥ 50 years old. The average annual incidence rate of HZ was 3.43/1,000 among people aged ≥ 50 years old in 2010–2012. Cumulative incidence and average annual incidence rate increased with age: the cumulative incidence of HZ among people aged ≥ 80 years old was 3.34 times of that among 50- years old (52.3/1000vs15.7/1,000); average annual incidence rate rises from 2.66/1,000 among 50- years old to 8.55/1,000 among 80- year old. Cumulative incidence and average annual incidence rate for females were higher than that for males (cumulative incidence, 26.5/1000vs18.7/1,000; annual incidence rate, 3.95/1000vs2.89/1,000). Cumulative incidence and average annual incidence rate in urban were higher than in rural (cumulative incidence, 39.5/1000vs 17.2/1,000; annual incidence rate, 7.65/1000vs2.06/1,000). The hospitalization rate of HZ was 4.53%. And with the increase of age, the rate has an increasing trend. HZ costs 945,709.5 RMB in total, corresponding to 840.6 RMB per patient with a median cost of 385 RMB (interquartile range 171.7–795.6). Factors associated with cost included the first onset year, area, whether hospitalized and whether sequelae left.

**Conclusion:**

Incidence rate, complications, hospitalization rate and average cost of HZ increase with age. We recommend that the HZ vaccinations should target people aged ≥50 years old if Zoster vaccine is licensed in China.

## Introduction

Herpes zoster (HZ), a disease characterized by clusters of blisters along the areas innervated by sensory nerve, is caused by varicella-zoster virus (VZV). The disease is often accompanied by severe pain that negatively impact the quality of life. The incidence of HZ correlates with increasing age, especially people after 50 years old [1–2]. It is estimated that the risks of occurring HZ in people previously infected with varicella are 10%-30% and the severity also increases with age [3–7].The annual incidence of HZ in the year 2013, 2012 and 2011 in Guangdong, China was 5.8, 3.4 and 4.1 per 1000 person-years, respectively[8].

Zoster vaccine is an effective method to prevent HZ. A few countries have introduced the Zoster vaccine into the national immunization program since it was licensed. The Zoster vaccine was licensed for people at least 50 years of age who had not have prior HZ for the prevention of HZ in Australia and Europe, while target age is at least 60 years of age in the US [9–12]. There is rare surveillance data in China on HZ due to it is not a notifiable disease. In addition current available data in China are mostly focused on treatments, only one study [8] on epidemiology and incidence, and no data on economic burden is available, especially community and population based data.

Developing an effective immunization strategy against HZ should base on the baseline data, such as epidemiology of HZ in target population, incidence and cost of HZ etc. Therefore we conducted this community based, retrospective study with objectives to understand incidence and economic burden of HZ in selected communities among people aged ≥50 year olds, so as to provide rationale for evidence based immunization strategy for HZ vaccination in China, as well as to provide baseline data for future similar studies.

## Materials and Methods

### Study sites and subjects

One township from each province of Jiangsu, Heilongjiang, Jiangxi and Hebei (as representative of rural areas) was selected; and one community from Shanghai (as representative of urban areas) was selected to conduct the retrospective study. The study was conducted from May 2013 to May 2014, targeting people aged ≥50 years old who developed HZ prior to the study. This study was approved by the ethical review committee of Chinese center for disease control and prevention (the approve number: 201313). All subjects signed the informed consent before they were recruited in the survey.

### Data collection and calculation of the incidence rate

#### Demographic information

Population register data from community residency committee (or village), or statistics from local public security department were used to collect demographic information for people aged ≥50 years old.

#### Case searching

We set up investigation team comprised of staff from local center for disease control and prevention, staff from local community health service center (or village doctors), and staff from community resident committees (or village committee). The team carried out a house to house census survey to collect HZ patient information, including all the self-reported HZ diagnosed by doctor. Thereafter the investigators carried out a face-to-face interview for the patients to collect detailed information on HZ disease.

#### Calculation of incidence rate

cumulative incidence rate=(number of people with prior HZ)÷(number of targeted people)×1000÷1000

Annual incidence rate=(number of people with HZ onset in a certain year)÷(number of targeted people)×1000÷1000

### Data Collection Method and Contents

We conducted a face to face interview using a uniformed questionnaire to collect information on basic demographic information, clinical manifestations, treatment information and economic burden of HZ patients. The clinical manifestations, treatment information and the cost in hospital of patients from the urban area were mainly collected from health care records and the receipts of payments for medical treatment. The same information of outpatients who had an onset date within 2 years and all the inpatients from rural area was mainly collected via reviewing the hospital outpatient logs / inpatient records and the reimbursement records from New Rural Co-financing Medical System and the Health Care Insurance System. The information of the remaining outpatients from rural area as well as other expense of all patients were collected via the payment receipts and memory provided by the subjects.

#### Contents of basic demographic information

The basic information included gender, date of birth and location.

#### Contents of clinical manifestations

We collected the detailed clinical manifestations for the HZ occurred first time and the second recurrence, the information included the date of onset, age when HZ onset, duration of rash, locations of the herpes, how long the pain lasted, whether sequela occurred and the classification of the severity. If the HZ reoccurred more than two times, we didn’t collect the later manifestations and just asked how many times the HZ occurred for the same patient.

#### Contents of treatment information

Treatment information included the total numbers of outpatient visit, the hospital level of the first 3 visits, whether to hospitalize, if hospitalized, the hospital level of each hospitalization for the first 3 times, the total days of hospitalization, the course and the outcome of HZ.

#### Contents and calculation of the cost

Contents of cost data: Cost information included: 1) outpatient expenses; 2) hospitalization expenses; and 3) other expenses which include the cost of Over-The-Counter Drug, transportation cost due to seek medical service, productivity loss (caring for the patient), and other costs considered to be associated with the disease.

Discount rate: The discount rate was used to adjust the cost in variable years. Consumer Price Indices (CPI), obtained from the website of China National Bureau of Statistics from 1951 to 2013 in China, was used to calculate the annual average discount rate as follows [13].

$$\text{Discount rate}=(\sum_{\text{year}=1951}^{2013}\text{CPI})÷(\text{N}+1)-1=0.036$$

N = 2013–1951 = 62, the average discount rate was 0.036, we used 0.04 as the average discount rate in this study.

Conversion of cost in different years: Based on the price of 2013, the cost occurred in other years is converted into 2013 price.

$$\text{year specific cost}=(\text{cost in ​ }2013)÷{\left(1+\text{discount rate}\right)}^{\left(\text{N-1}\right)}$$

N=2013−onset year

### Statistical method

EPI Data 3.1 was used to set up database, and double data entry was used to guarantee the quality. Statistical Product and Service Solution (SPSS version 17.0) was used for data analysis. Chi square was used to test qualitative data, with relative risk (RR) and 95% confidence interval (95%CI) calculated. ANOVA, t test and multiple linear regression were used to test quantitative data. First type error level was set to be 0.05.

## Results

### Incidence of HZ

The total target subjects aged ≥50 years old in study areas were 49,721 in our study, and 1126 people had developed HZ, the cumulative incidence rate was 22.6/1,000. Cumulative incidence increased with age, the highest incidence was among people aged ≥ 80 years old which was3.34 times higher than that among people aged 50- years old (52.3/1000vs 15.7/1,000). There were 461 male patients and 665 female patients with a male to female ratio of 1:1.44. Cumulative incidence rate in female was higher than that in male (26.5/1000 vs. 18.7/1,000, RR = 1.41, 95% CI = 1.26–1.59). There were 648 patients in rural areas, and 478 in urban areas. Cumulative incidence rate was higher in urban areas than that in rural areas (39.5/1000 vs17.2 /1,000, RR = 2.29, 95%CI = 2.04–2.57) (Table 1).

**Table 1:** Cumulative incidence and epidemiological characteristics of HZ in people aged ≥50 years old.

Variable	Population	Patient Number	Cumulative incidence (/1,000)	RR	95%CI
Age group (years)					
50-	21571	338	15.7	Ref	-
60-	16896	389	23.0	1.47	1.27–1.70
70-	8135	236	29.0	1.85	1.57–2.18
80-	3119	163	52.3	3.34	2.78–4.01
Gender					
Male	24598	461	18.7	Ref	-
Female	25123	665	26.5	1.41	1.26–1.59
Area					
Rural	37607	648	17.2	Ref	-
Urban	12114	478	39.5	2.29	2.04–2.57
Total	49721	1126	22.6		

There were 144, 159 and 208 HZ patients in 2010, 2011, 2012 respectively, with an incidence rate of 2.90/1,000, 3.20/1,000 and 4.18/1,000 among people aged ≥ 50 years old. The average annual incidence rate was 3.43/1,000 for year 2010–2012. During the three years, average age-specific incidence rate increased with age, from 2.66/1,000 in 50- years old to 8.55/1,000 in 80- years old. Average annual incidence rate was higher in female than in male (3.95/1000 vs 2.89/1,000) and higher in urban areas than in rural areas (7.65/1000 vs 2.06/1,000) (Table 2).

**Table 2:** Incidence rate (/1,000) of HZ among people aged ≥ 50 years old from 2010 to 2012.

Variable	Population	2010		2011		2012		Mean annual incidence rate
		Patient Number	Incidence rate	Patient Number	Incidence rate	Patient Number	Incidence rate	
Age group (years)								
50-	21571	37	1.72	66	3.06	69	3.20	2.66
60-	16896	54	3.20	41	2.43	70	4.14	3.26
70-	8135	26	3.20	33	4.06	35	4.30	3.85
80-	3119	27	8.66	19	6.09	34	10.90	8.55
Gender								
Male	24598	63	2.56	60	2.44	90	3.66	2.89
Female	25123	81	3.22	99	3.94	118	4.70	3.95
Area								
Rural	37607	61	1.62	81	2.15	91	2.42	2.06
Urban	12114	83	6.85	78	6.44	117	9.66	7.65
Total	49721	144	2.90	159	3.20	208	4.18	3.43

### Characteristics of HZ

#### First onset age

In total 1,125 patients out of 1,126 reported the age of first onset, with the median age of 59 years old and interquartile of 52–68 years old. Cumulative incidence rate of HZ before 50 years old was 3.5/1,000, and increased with age, to 8.0/1,000, 10.7/1,000, 14.4/1,000 and 27.6/1,000 for 50-, 60-, 70-, and 80- years old age respectively (Table 3).

**Table 3:** Cases and cumulative incidence rates of HZ according to first onset age in people aged ≥ 50 years old.

First Onset age*	Patient Number					Cumulative Incidence Rates (/1,000)				
	50-	60-	70-	80-	Sub-total	50-	60-	70-	80-	Sub-total
<50	107	44	13	11	175	5.0	2.6	1.6	3.5	3.5
50-	231	141	21	7	400	10.7	8.3	2.6	2.2	8.0
60-		204	92	6	302		12.1	11.3	1.9	10.7
70-		-	109	53	162			13.4	17.0	14.4
80-		-	-	86	86				27.6	27.6

* One patient didn’t report onset time.

#### Sequelae occurrence

About 16.6% (187/1,126) of HZ had sequelae left after HZ, including neuralgia (182), followed by erythema (15), papulovesicle (9), and blister (4), etc. The frequency of sequelae increased with age (Table 4).

**Table 4:** Age specific sequelae occurrence in HZ patients aged ≥ 50 years old.

Onset age*	Patient Number	Patientswith sequelae	Percentage (%)	Trends χ2	*P*
<50	175	27	15.4	8.486	0.0036
50-	400	54	13.5		
60-	302	48	15.9		
70-	162	36	22.2		
80-	86	22	25.6		
Total	1126	187	16.6		

* One patient didn’t report onset time.

#### Recurrence

On average 2.8% (32/1,126) of HZ reported a second recurrence, 0.2% (2/1126) reported a third recurrence. No patients reported more than 3 times of recurrence.

### Hospitalization rate of HZ

In total 4.53% (51/1,126) of HZ were admitted into hospital for treatments. The average length of hospital stay was 10 days (range: 3–51 days), with a median of 12.7 days based on 50 patients with complete information. There was statistical difference in hospitalization rates among gender and area: the rate for male higher than that for female, and higher in rural than that in urban. (Table 5).

**Table 5:** Hospitalization rates among HZ patients aged ≥ 50 years old and associated factors: logistic regression analysis.

Variable	Parameter estimation	Standard error	Wald-Chi-Square	*P*	OR	*95%CI*
Age	0.1688	0.3138	0.2894	0.5906	1.184	0.640–2.190
First Onset age	0.1957	0.3040	0.4146	0.5197	1.216	0.670–2.207
First onset year	0.4502	0.2878	2.4465	0.1178	1.569	0.892–2.758
Gender	-0.7145	0.2947	5.8782	0.0153	0.489	0.275–0.872
Area	1.8687	0.4116	20.6135	<0.0001	6.480	2.892–14.517

### Cost of HZ

Cost data were collected from 1,125 HZ patients with only 1 missing because the discount rate couldn’t be calculated resulting from his being unable to remember the onset date. The cost for outpatient was not available for 18 out of the 1,125 patients, and not available for 2 inpatients.

The total cost of enrolled HZ patients was 945,709.5 RMB, with a mean of 840.6 RMB per patient, and a median cost of 385 RMB (interquartile: 171.7–795.6) (Table 6). After log conversion, the univariate analysis showed that cost of HZ was related with area, first onset age, first onset year, hospitalization or not, and whether there were any sequelae (Table 7). Multivariate linear regression analysis found that the factors associated with the cost include first onset year, area, hospitalization or not, and whether there were any sequelae (Table 8).

**Table 6:** Cost of HZ among people aged ≥50 years old (RMB).

Variable	Total cost	Number of patients	Average	Median	25% percentile	75% percentile	Minimum	Maximum
Outpatient expenses	601997.5	1107^#^	543.8	336	135.2	649.6	0	13312
Hospitalization expenses	220618.5	49^%^	4502.4	3260.4	1161.7	5715	136.6	30000
Other expenses	123093.6	1125	109.4	0	0	16.6	0	4401
Total	945709.5	1125	840.6	385	171.7	795.6	0	30002

^#^ Cost of outpatient was not available for 18 patients.

^%^ Cost of inpatient was not available for 2 patients

**Table 7:** Cost of HZ among people aged ≥ 50 years old and associated factors (RMB): Univariate analysis.

Variable	Number of patients	Total cost	Mean	Log Mean	F or t	*P*
Area						
Rural	647	519520.66	802.97	257.04	104.32	<0.0001
Urban	478	426188.87	891.61	588.84		
Gender						
Male	461	402035.08	872.09	363.08	0.18	0.6686
Female	664	543674.45	818.79	371.54		
Age groups (years)						
50-	338	227836.05	674.07	331.13	2.29	0.0773
60-	389	293567.79	754.67	346.74		
70-	235	267454.04	1138.1	407.38		
80-	163	156851.64	962.28	446.68		
First Onset age						
<50	175	110921.31	633.84	165.96	20.67	<0.0001
50-	400	270546.53	676.37	363.08		
60-	302	276600.07	916.89	416.87		
70-	162	213582.86	1318.41	562.34		
80-	86	73758.77	857.66	537.03		
First Onset year						
<1990	71	26207.31	369.12	69.18	46.27	<0.0001
1990-	53	36286.74	684.66	208.93		
2000-	375	324018.12	864.05	331.13		
2010-	626	559197.36	893.29	478.63		
Hospitalized						
Yes	51	286358.83	5614.88	3890.45	179.39	<0.0001
No	1074	659350.7	613.92	323.59		
Sequelae						
Yes	185	226029.38	1221.78	660.69	7.54	<0.0001
No	940	499061.64	530.92	302.00		

**Table 8:** Cost of HZ among people aged ≥ 50 years old and associated factors (RMB): Multivariate analysis.

Variable	Parameter estimation	Standard error	t	*P*
First Onset age	0.02780	0.01489	1.87	0.0622
First onset year	0.14725	0.02178	6.76	<0.0001
Sequelae	-0.30710	0.04071	-7.54	<0.0001
Hospitalization	-0.54296	0.07629	-7.12	<0.0001
Gender	0.05173	0.03080	1.68	0.0933
Area	-0.27258	0.03427	-7.96	<0.0001

F = 56.06, *P*<0.0001; R^2^ = 0.2393, adjusted R^2^ = 0.2351

## Discussion

HZ is a common viral disease, due to viruses reactivated when the VZV specific immunity declines [14–15]. We found that cumulative incidence of HZ was 22.6/1,000 (17.2/1000 in rural areas and 39.5/1000 in urban areas) among people aged ≥50 years with an average annual incidence rate of 3.43 / 1,000 (2.06/1000 in rural areas and 7.65/1000 in urban areas), which is similar to that in Guangdong, China [8] and that in North America, Europe and Asia-Pacific (3-5/1000) [16]. The average annual incidence rate in urban areas reported by our study is similar to that in Taiwan (>8.36/1,000) [17], Australia (9.67/1,000) [18], The United States (8.46/1000) [19] and South Korea (10/1,000) [20]. Both cumulative incidence and annual incidence rate of HZ increased with age, possibly related to the decline in immunity with age, which is consistent with reported abroad [1–2, 21–22]. We also found a higher cumulative incidence and annual incidence rate in female than that in male, consistent with some domestic and abroad studies [8,19–20, 22–26], although some studies [27–28] reported a non-statistical difference in incidence between female and male. Therefore, further researches are needed to understand whether a difference between genders really exists and if so, the mechanisms. Cumulative incidence and annual incidences vary among different regions. We found a lower incidence in rural areas than that in urban areas, with contributors to be further clarified. We also found a similar recurrent rate of 3% (34/1126), similar to that reported by Jie Fang (2.72%)[29]; and a hospitalization rate of 4.53%, similar to reported in Taiwan (>4.14% among people aged ≥60 years old) [17], but lower to that in Guangdong, China [8].

This study showed that the mean cost per HZ case was 840.6 RMB, with a median of 385 RMB. The majority of cost was direct treatment cost related to outpatient (average of 543.8yuan) and hospitalization costs (average of 4502.4yuan). The indirect cost related HZ was relatively low. Cost was higher for inpatients than for outpatients, and higher for patients with sequelae than those without sequelae.

There were 338 million people aged ≥50 years old in China (183 million in rural areas, and 155 million in urban areas) according to census in 2010 [30]. Based on cumulative incidence rate of HZ in rural areas and urban areas, it is projected that there were 9.27 million people who had suffered from HZ aged ≥50 years old across China, with a total cost of 7.793 billion RMB. Based on the average annual incidence rate in rural China and urban China in 2010–2012, we estimate that there are 1.563 million new HZ cases every year with a total cost of about 1.314 billion RMB across China.

In addition to the direct economic burden caused by HZ, the quality of life of patients is negatively influenced due to the severe pain caused by the disease, with physical (e.g., fatigue, weight loss, insomnia), psychological (e.g., difficulty in concentrating, depression), social (e.g., decreased social activities, change of social role) and functional (e.g., dressing, mobilization) influenced. These effects are particularly severe for elderly patients with HZ [31]. In the US, the postherpetic neuralgia is the third common cause of chronic neuropathic pain [32]. Therefore, it is necessary to consider the impact of disease on the quality of life of the patients in addition to the direct economic burden when HZ immunization strategy is developed.

The age specific incidence rate, sequelae, hospitalization rate, average cost of HZ all increase with age, therefore vaccination targeting younger age groups would be the most cost effective. It is recommended that Zoster vaccine is used for people aged ≥60 years old in US [12], and it is recommended that the Zoster vaccine is indicated in people aged ≥50 years old in Australia [10] and in Europe [11]. Considering local epidemiology of HZ and immunization policy abroad, based on the immune response observed in vaccinees aged 50–59 years comparable to that observed in vaccinees 60 years or older [33], we recommend that the Zoster vaccine is used for people aged ≥50 years old when it is available in China in the future and the long term protection of the vaccination also should be monitored in order to determine if the potential booster dose is necessary.

There are a few limitations in this study: 1) Memory bias. In total 11.1% of the HZ occurred before 2000, resulting in less reliable incidence and cost due to the only information source is memory. 2) The data on mortality and fatality couldn’t be obtained because since the retrospective nature. 3) Study sites were not selected randomly and subjects recruited were people aged ≥ 50 years old, therefore the results can only represent the population aged ≥ 50 years old in selected sites and the generalization to the population aged ≥ 50 years old in other areas should be with caution. However, because the provinces selected are representative of east, middle and west China, and rural and urban areas were selected, this study can partly reflect the incidence and cost of HZ in people aged ≥ 50 years old. This study can provide baseline information for future studies with representative sampling methods.

## Supporting Information

S1 Dataset Minimum database. (XLSX)

S1 File Declaration of variables in minimum database. (DOCX)
