# Supplementary material for: Disease Burden Due to Herpes Zoster among Population Aged ≥50 Years Old in China: A Community Based Retrospective Survey
Source: PLoS One. 2016 Apr 7;11(4):e0152660. doi: 10.1371/journal.pone.0152660 (PMC4824529; doi:10.1371/journal.pone.0152660)
Supplement: S1 File — (DOCX) [file pone.0152660.s002.docx]

**The declaration of variables in minimum database**

| Variables | Declaration |
| --- | --- |
| gender | 1:male 2:female |
| type | Location 1:urban 2: rural |
| fbdate | Date of first onset |
| sequelae1 | Whether had sequalae after first onset.1:yes 2:no 3:unknown |
| hb1 | Erythema after first onset which was one manifestation of sequalae. 1:yes 2:no 3:unknown |
| qpz1 | Papulovesicle after first onset which was one manifestation of sequalae.. 1:yes 2:no 3:unknown |
| sp1 | Blister after first onset which was one manifestation of sequalae. 1:yes 2:no 3:unknown. |
| sjt1 | Neuralgia after first onset which was one manifestation of sequalae. 1:yes 2:no 3:unknown. |
| second | Whether the second onset happened? 1:yes 2:no 3:unknown |
| sequelae2 | Whether had sequalae after second onset.1:yes 2:no 3:unknown |
| hb2 | Erythema after second onset which was one manifestation of sequalae. 1:yes 2:no 3:unknown |
| qpz2 | Papulovesicle after second onset which was one manifestation of sequalae.. 1:yes 2:no 3:unknown |
| sp2 | Blister after second onset which was one manifestation of sequalae. 1:yes 2:no 3:unknown. |
| sjt2 | Neuralgia after second onset which was one manifestation of sequalae. 1:yes 2:no 3:unknown. |
| total | The total number of onset |
| seridig | The classification of severity |
| number | Times of outpatient visit |
| totalfee | The total cost of outpatient visit |
| hspitaliz | Whether to hospitalize.1:yes 2:no |
| totalday | Days of hospitalization |
| totalcost | The total cost of hospitalization |
| drug | The cost of Over-The-Counter Drug |
| transport | Transportation cost due to seek medical service |
| othercost | Other costs considered to be associated with the disease |
| people | Number of people caring for patients |
| meanday | Days to care for patients |
| salary | The salary of people caring for patients |
| dctime | Date of survey |
